# Quantified forces between HepG2 hepatocarcinoma and WA07 pluripotent stem cells with natural biomaterials correlate with *in vitro* cell behavior

**DOI:** 10.1038/s41598-019-43669-7

**Published:** 2019-05-14

**Authors:** Riina Harjumäki, Robertus Wahyu N. Nugroho, Xue Zhang, Yan-Ru Lou, Marjo Yliperttula, Juan José Valle-Delgado, Monika Österberg

**Affiliations:** 1 Department of Bioproducts and Biosystems, School of Chemical Engineering, Aalto University, FI-00076 Aalto, Finland; 2 Division of Pharmaceutical Biosciences, Faculty of Pharmacy, University of Helsinki, FI-00014 Helsinki, Finland; 3 Department of Pharmaceutical and Pharmacological Sciences, University of Padova, I-35131 Padova, Italy

**Keywords:** Pluripotent stem cells, Biomaterials - cells, Applications of AFM

## Abstract

*In vitro* cell culture or tissue models that mimic *in vivo* cellular response have potential in tissue engineering and regenerative medicine, and are a more economical and accurate option for drug toxicity tests than animal experimentation. The design of *in vivo*-like cell culture models should take into account how the cells interact with the surrounding materials and how these interactions affect the cell behavior. Cell-material interactions are furthermore important in cancer metastasis and tumor progression, so deeper understanding of them can support the development of new cancer treatments. Herein, the colloidal probe microscopy technique was used to quantify the interactions of two cell lines (human pluripotent stem cell line WA07 and human hepatocellular carcinoma cell line HepG2) with natural, xeno-free biomaterials of different chemistry, morphology, and origin. Key components of extracellular matrices –human collagens I and IV, and human recombinant laminin-521−, as well as wood-derived, cellulose nanofibrils –with evidenced potential for 3D cell culture and tissue engineering– were analysed. Both strength of adhesion and force curve profiles depended on biomaterial nature and cell characteristics. The successful growth of the cells on a particular biomaterial required cell-biomaterial adhesion energies above 0.23 nJ/m. The information obtained in this work supports the development of new materials or hybrid scaffolds with tuned cell adhesion properties for tissue engineering, and provides a better understanding of the interactions of normal and cancerous cells with biomaterials in the human body.

## Introduction

Cells and their extracellular matrix (ECM) have a constant interplay in various ways. These interactions are crucial for successful cell culture *in vitro* and normal cell behavior *in vivo*^1–4^. In tissues, different ECM macromolecules are often integrated to form complex tissue-specific structures. ECM proteins are primarily composed of two general classes of macromolecules: fibrous proteins, such as collagens and elastin, and glycoproteins, including laminin, vitronectin, fibronectin, and proteoglycans. Together these materials form a physical, chemical, and biological three-dimensional (3D) environment for cells in tissues. In *in vitro* cell cultures, the rigidity, topography, and chemistry of the matrix biomaterials affect cell proliferation, viability, and differentiation^5–7^. A good *in vitro* cell culture model mimics the natural *in vivo* environment by combining biomaterials with cells and soluble factors. Unfortunately, it is still not fully understood how the properties of the biomaterials affect their interactions with cells because quantitative information on the interactions between cells and different biomaterials is still scarce^8^.

Materials traditionally used for generating new tissue models have many drawbacks. For example, Cultrex^®^ or the widely used Matrigel^®^, secreted by mouse sarcoma cells, have great variability from batch to batch and danger of xenobiotics. Synthetic materials often need the use of toxic cross-linking agents to form 3D scaffold structure^9^. Therefore, new suitable materials and chemistry for cell culture scaffolds are needed, but they are usually selected based on trial-and-error tests and the reasons for their suitability quite often remains unknown. One example of a novel cell culture material is chemically unmodified, plant-derived, and thus xenobiotic-free, hydrogel from cellulose nanofibrils (CNF, also known as nanofibrillated cellulose, NFC). It has excellent physical properties for cell culture applications, and fibrous morphology resembling natural extracellular matrix^10^. Unmodified, plant-derived CNF hydrogel has been shown to be suitable for 3D cultures of various cell lines, allowing them to form spheroids, small tissue-like cell aggregates^11,12^. Even delicate human pluripotent stem cells (hPSCs) were cultured in the CNF hydrogel and remained undifferentiated for long periods of time^13^. Moreover, this unmodified CNF is biocompatible and does not cause an immune reaction *in vivo*^14^, and has been used as wound dressing in clinics^15^. Nevertheless, the specific interactions between CNF and the cultured cells remain unknown.

The underlying mechanisms of interactions between cells and biomaterials have drawn special attention. The discovery of various subtypes of integrins and other cell surface proteins and molecules have shed some light on the molecular mechanisms responsible for cell-biomaterial interactions. This knowledge has now been used for tissue engineering purposes; for instance, Kanninen *et al*. detected the components of ECM from their target tissue and created accordingly a highly defined engineered cell culture scaffold to trigger stem cell differentiation into this tissue^16^. The interactions between cells and biomaterials occur at the nanoscale and in physiological solutions, which limits the suitable methods for their detection. Washing assay^17^ and flow chambers or spinning disc devices^18–21^ have been applied to study cell-biomaterial interactions. Nevertheless, it is difficult to get quantitative and detailed information about these interactions with those methods. To determine the interaction forces and binding kinetics of cells and substrates quantitatively, various single cell force spectroscopy (SCFS) assays have been used. While micropipettes^22^, magnetic-^23–25^, and optical tweezers^26,27^ have disadvantages either because of low force resolution or narrow range of detectable forces (from 10 pN to 1 nN)^28^, atomic force microscope (AFM)-based force spectroscopy offers a broader range of detectable forces from 10 pN to 100 nN. It furthermore provides accurate temporal (~0.1 s to >10 min) and spatial (~1 nm to ~100 µm) control during the adhesion measurement at physiological conditions^28^. AFM force spectroscopy has been applied to study the interactions between biomaterials and human cells such as myeloid leukemia^29^, breast cancer^30^, and embryonic kidney cells^31^. These measurements were performed by attaching a cell to the AFM cantilever and then probing against the materials attached to the substrate. This method is not suitable for delicate cell lines that cannot survive as single cells. In addition, it is also difficult to control the cell viability during the measurements with this setup. To ensure the viability of sensitive cells we employed the opposite setup, attaching a small micrometer-sized glass sphere coated with the materials of interest to the AFM cantilever and culturing the cells on the substrate. This set-up, called AFM-colloidal probe technique or colloidal probe microscopy (CPM)^32^, has been widely used to study interactions between various materials, but seldom for cell interaction studies^33^. This method allows faster and gentler approach to very sensitive cells compared to SCFS.

In this study, we explore for the first time the interactions of selected biomaterials with a human pluripotent stem cell (hPSCs) line WA07 and a human hepatocellular carcinoma cell (HCC) line HepG2. The hPSCs are delicate cells that can be successfully cultured on only a few materials^34^. They can proliferate as stem cells efficiently and differentiate into any kind of cell type of the human body. In other words, they could serve as a limitless bank of all human cells. These cells provide great potential for drug testing, disease modeling, and tissue engineering. On the other hand, the HepG2 cell line is a typical carcinoma cell line that survives well in various environments. Hepatic cells are important in the drug development process since the liver is the main detoxifying organ in the human body and mainly responsible for drug metabolism and drug-drug interactions. Hence, HepG2 cells are widely used for drug development and toxicity testing. Furthermore, the interactions between cancer cells and ECM proteins are particularly important in cancer metastasis and tumor progression^35–45^. A better knowledge of them could help us to find new methods for cancer treatments.

All the biomaterials used in this study are xenobiotic free and thus suitable for regenerative medicine. They have previously been used with either or both of these cell lines for creating tissue models. Human collagen I (Col I) and human collagen IV (Col IV) have been used as matrices for HepG2 cells^46,47^, and CNF hydrogel^11,13^ and human recombinant laminin-521 (LN-521) have recently been explored for hPSC and HCC cultures^16,48,49^. Collagen is the most abundant ECM protein in the human body and can be found in various connective tissues^50,51^. Col I is structurally composed of three primary chains that form a semi-rigid helical structure. In contrast to Col I, type IV collagen forms a net-like structure and is primarily found in the basement membranes^52^. Col IV interacts with laminins, which are the major non-collagenous component of the basal lamina. Laminins are heterotrimeric glycoproteins with a cruciform shape that bind to other components of ECM and cell membranes^53,54^. On the other hand, a CNF hydrogel has a physical environment resembling the natural fibrillar ECM^10^, but its chemical nature differs significantly from the other materials; CNF is made of cellulose that is a polysaccharide formed from glucose units, whereas ECM proteins consist of chains of various amino acids. It is not yet fully understood how these morphology and chemical nature variations affect the cell interactions, even though that is an area of intensive research.

This study aims to understand the nature of the cell-biomaterial interactions in order to develop better *in vitro* tissue models. To achieve this, we have applied CPM to explore the interactions between two different cell lines with high impact in drug testing and tissue engineering (hPSCs WA07 and HCCs HepG2) and some relevant biomaterials (Col I, Col IV, LN-521, and CNF) at nanoscale systematically. To the best of our knowledge, the interactions of CNF and laminin-521 with any cells have not been measured by CPM before, and detailed quantification of the interactions of hPSCs with different biomaterials has not been carried out so far. The information obtained from direct surface force measurements could support the development of new 2D and 3D scaffolds with tuned cell adhesion properties, and it could also be valuable for better understanding the behavior of normal and cancerous cells.

## Experimental Section

### Preparation of biomaterial solutions and dispersions

The biomaterial solutions and dispersions were prepared as described previously by us^55^ based on the protocols provided by Goffin *et al*.^56^ for collagens and Valle-Delgado *et al*.^57^ for CNF. Briefly, the human collagen type I (Collagen from human placenta, C7774-5MG, Sigma) and human collagen type IV (Collagen from human placenta, C7521-5MG, Sigma) solutions were dissolved with acetic acid to a final collagen concentration of 1 mg/mL at pH 3. Collagen solution aliquots were prepared sterile, stored at −20 °C, and thawed and sonicated in ice prior to use. Sterile human recombinant laminin-521 solution (LN521-02, 10 mg/ml, Biolamina) was diluted in sterile Dulbecco’s phosphate buffered saline with calcium and magnesium (1 × DPBS+, 14040-133, Gibco^TM^) into a final concentration of 10 µg/ml. A 1.35 g/l dispersion of CNF with 0.88% dry matter content was prepared by diluting plant-derived, sterile CNF hydrogel prepared without chemical modification (GrowDex^®^, UPM-Kymmene; additional information on composition and low surface charge is provided by Lou *et al*.^13^) in deionized water followed by ultrasonication at 25% amplitude for 1 min using a Branson sonifier S-450 D (Branson Corp., Danbury, CT). The CNF dispersion was then centrifuged at 8000 × g for 30 min at room temperature (RT) with an Eppendorf centrifuge 5804R (Eppendorf AG, Hamburg, Germany) and the supernatant fraction with the finest CNF fibrils was collected.

### Preparation of colloidal probes

Two different types of tipless silicon cantilevers, CSC38/No Al and NSC36/Cr-Au (MikroMasch, Wetzlar, Germany) with normal spring constants in the range 0.01–0.36 N/m and 0.1–4.6 N/m, respectively, were used in the study. Glass microspheres with diameter of 15–40 µm (Polysciences, Inc., Warrington, PA) were used as colloidal probes. The colloidal probes had similar size as the cells used in this work (between 10 and 30 µm for WA07 and about 20 µm for HepG2)^58,59^. The glass microspheres were glued at the free end of the cantilevers with the aid of a motorized PatchStar micromanipulator (Scientifica, Uckfield, UK) and an optical adhesive glue (Norland Products, Inc., Cranbury, NJ) and cured under UV light (wavelength of 365 nm) for 15 min.

Biomaterials were adsorbed onto the probes with different techniques as presented by Nugroho *et al*.^55^. The glass microspheres to be coated with collagens were previously cleaned in a piranha solution for 15 min, rinsed with MilliQ water, and silanized with (3-aminopropyl) triethoxysilane (APTES) prior to coating. The silanization took place in 5% (v/v) APTES solution in ethanol for 45 min at RT, followed by thorough rinsing with ethanol and overnight drying.

Collagen-coated colloidal probes were obtained by spin-coating Col I and Col IV solutions on the cantilevers with the attached microspheres. The spin-coating was carried out at 1000 rpm for 40 s using a Laurell spin-coater WS-650SX-6NPP-Lite (Laurell Technologies Corp., North Wales, PA). The coated probes were dried overnight, rinsed with MilliQ water, dried again and stored at RT until use.

Laminin-coated colloidal probes were prepared just before use by immersing the colloidal probes in drops of sterile LN-521 solution deposited on a polytetrafluoroethylene film. Laminin adsorption took place for about 2 h at RT in a humidified chamber. The laminin-coated colloidal probes were rinsed with 1 × PBS+ and immediately used in the force experiments.

CNF-coated colloidal probes were also prepared by adsorption. Firstly, polyethyleneimine (PEI, Aldrich) was adsorbed by immersing the colloidal probes in drops of 2.5 mg/mL PEI for 10 min followed by rinsing with MilliQ water. The probes were then immersed in drops of CNF dispersion for another 10 min. The CNF-coated probes were finally rinsed with MilliQ water and dried under flowing nitrogen.

Control force experiments were carried out with HepG2 cells and uncoated, PEI-coated, and APTES-coated glass probes, which were prepared similarly as described above.

### Preparation of film substrates

Plastic coverslips (Sarstedt, 83.1840.002) were coated with the different biomaterials used in this work to see the effect of the material on cell behavior *in vitro*. The coverslips were coated following similar protocols as for colloidal probes. Briefly, laminin-coated coverslips were prepared by immersing the coverslips in laminin solutions for two hours at RT. After laminin adsorption, the coverslips were kept in 1 × DPBS+ at +4 °C for up to two weeks. Coverslips exposed to 2.5 mg/mL PEI solution for 10 min and rinsed with MilliQ water were coated with CNF by spin-coating at 4000 rpm for 1 min. Collagen coatings were prepared by adding a few drops of collagen solution on top of APTES-coated coverslips and further spin-coated at 1000 rpm for 40 s. The collagen-coated coverslips were dried overnight and rinsed with MilliQ water before use.

### Cell maintenance

The culture of the human hepatocellular carcinoma HepG2 cells from ATCC (HB-8065) was performed in 75 cm^2^-cell culture flasks in DMEM with high glucose and pyruvate content (Gibco, 41966-029) medium supplemented with 10% fetal bovine serum (Gibco, 10270-106). Cells were passaged twice a week at a ratio of 1:5 by using TrypLE^TM^ Express (Gibco^TM^, 12604-021). The human embryonic stem cell line WA07 (WiCell) was cultured on Matrigel-coated 6-well plates (Matrigel basement membrane matrix growth factor reduced, BD Biosciences, 356230, 0.5 mg per one 6-well plate). The mTeSR™1 medium (STEMCELL™ Technologies, 05850) was changed daily. The Matrigel coating was prepared by incubating diluted Matrigel solution for one hour at room temperature. Stem cells were passaged at a ratio of 1:4 to 1:6 when the confluency had reached 70–90%. After manual removal of differentiated cells, the stem cells were detached with Versene 1:5000 (Invitrogen, 15040-033). All the cell cultures were maintained at 37 °C in a humid atmosphere with 5% CO_2_.

### Cell culture for force measurements and biomaterial studies

For CPM experiments and as a control for biomaterial studies the HepG2 and WA07 cells were seeded on uncoated or Matrigel-coated plastic coverslips, respectively, kept on a 12-well plate. For the biomaterial experiments the detached cells were seeded to the plastic coverslips coated with the studied materials, Col I, Col IV, LN-521, and CNF, as explained above, to evaluate how well the cells grow on the studied biomaterials. The biomaterial films were sterilized with UV light for 20 min before cell seeding and three replicas for each system were performed. Cells were cultured on the test materials or control wells for 20 hours prior to the analysis. The number of seeded cells was always similar in each culture systems for both cell lines. The cells were allowed to recover from splitting for at least two days before AFM measurements.

### Atomic force microscopy

A MultiMode 8 AFM with a NanoScope V controller (Bruker, Santa Barbara, CA) was utilized to obtain high-resolution images of the colloidal probes in air using ScanAsyst^®^ mode and ScanAsyst-air probes (Bruker). Research NanoScope 8.15 or NanoScope Analysis 1.5 softwares (Bruker) were used for image analysis. The only image correction applied was flattening. Root mean square (RMS) surface roughness was calculated from 1 × 1 μm^2^ images.

### Force measurements by AFM-colloidal probe technique

Cell-biomaterial force measurements were conducted with a MultiMode 8 AFM equipped with a closed-loop PicoForce scanner (Bruker) in 1 × PBS+. All the force experiments were carried out at 37 °C (Bruker TAC Thermal Applications Controller, Santa Barbara, CA) in clean laboratory spaces. Prior to force measurements, the spring constants of the cantilevers were determined via the analysis of thermal vibration spectra and the application of Sader equation^60^. The deflection sensitivity was determined on a freshly cleaved mica surface.

**Figure 1:**
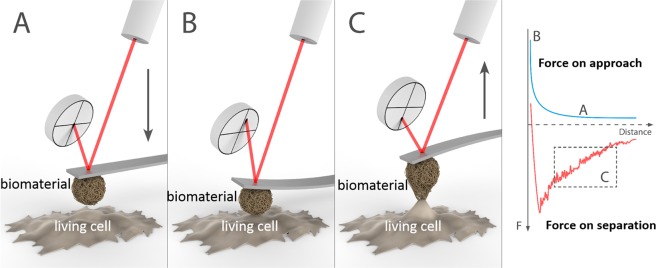
Schematic representation of the measurement of cell-biomaterial interaction forces by colloidal probe microscopy. A biomaterial-coated colloidal probe and a substrate with living cells are approached each other (**A**) until contact (**B**), and then they are retracted (**C**) until detachment. The interaction forces are quantified from the deflection of the cantilever, which is monitored with a laser and a photodetector. Figure prepared by Joel Wolff.

The cells on a plastic coverslip were washed twice with sterile 1 × PBS+ and mounted in the AFM liquid cell together with the biomaterial-coated colloidal probe. The system was allowed to equilibrate for 10 min in buffer prior to force measurements (Fig. 1). The interaction forces between probes and cells were recorded at 2 µm/s rate with at least 20 s lag between two consecutive approach-retraction cycles. Cells and probes were kept in contact for different times (1 s, 10 s, and 30 s) before retracting them to analyze the effect of the contact time on the cell-material adhesion. The highest contact time was fixed at 30 s for two main reasons: (i) longer contact times considerably limited the number of force curves that could be obtained within the time window where the cells were alive; and (ii) longer contact times increased the biomaterial-cell adhesion to the limit where the AFM scanner was not able to detach the colloidal probes from the cells. The force curves were triggered using relative trigger thresholds in order to get similar maximum applied forces for the different systems. The z-range was adjusted based on the strength of obtained force curves and varied between 8 and 14 µm. For each cell-material system, several force curves were captured using the same or different probes and cell plates within the same day or in different days to check the reproducibility of the measurements. The amount of force curves for each system is presented in the Supplementary Information (Tables S1 and S3) and varied between 10–48 for the cell-biomaterial interactions. The force curves were normalized by the radius of the colloidal probe. Cell-biomaterial adhesion energies were calculated by integrating the areas enclosed between the retraction force curves and the zero baselines. Pull-off forces were determined as the maximum adhesion forces (in absolute value) measured in the retraction force curves. Cell elasticity was estimated by fitting the approach force curves to the linearized Hertz model according to the equation^61^:

$${F}^{2/3}={(\frac{4E\sqrt{R}}{3(1-{\nu }^{2})})}^{2/3}\,\delta $$

where *F* is the measured force, E is the Young’s modulus of the cell, *R* is the radius of the colloidal probe, *δ* is the cell deformation (indentation), and *ν* is the Poisson ratio of the cell, which was assumed to be equal to 0.5 (Fig. S2).

### Cell morphology and cell viability

Cell growth, morphology, and viability were controlled during cell culture with a phase contrast microscope (Leica DM IL LED) with LAS EZ software (Leica DM 750 Microsystems, Switzerland). The cell viability during force experiments was controlled with a digital camera (uEye capture device filter with camera model UI148XLE-C, Obersulm, Germany) connected to the AFM instrument. Typically, cells started gradually to detach and die after 2 hours of experiments, so the measurement time was always kept under 2 h or 1.5 h for HepG2 and WA07 cells, respectively. In addition to the visual observation of the cell morphology commonly used in AFM force spectroscopy studies to monitor cell state, we also checked the cell viability after the force measurements by the Trypan Blue exclusion test with cell fixation for adherent cells, a protocol provided by Perry *et al*.^62^. Briefly, cells were dipped into trypan blue solution (1:5 dilution, Trypan Blue Solution, 0.4%, Gibco^TM^, 15250-061) and washed with 1 × DPBS solution. After fixation in 4% paraformaldehyde solution for 10 min the cells were washed twice and mounted on the objective glass with ProLong^®^ Gold Antifade reagent (Invitrogen, P36934). The imaging of the fixated cells was performed with a Leica phase contrast microscope (Leica DM750) with LAS EZ software.

### Statistical analysis

Root mean square (RMS) surface roughness values were average values analysed with Origin Pro software by using three different locations per image. Standard deviation was used to describe the error. Adhesion energy and maximum pull-off forces were calculated with the aid of Origin Pro software. The amount of force curves analyzed varied between 10 and 48 per measured system (Table S1). For controls, the number of force curves varied from 5 to 18 (Table S3). Forces were probed on at least two different locations. Mean values were calculated and standard error of mean was used to describe the error. The statistical difference between the mean values of two independent groups of adhesion energy or pull-off force data was estimated with Welch’s *t*-test. The data were considered significantly different for p ≤ 0.05.

## Results

### The morphology of biomaterial surfaces

**Figure 2:**
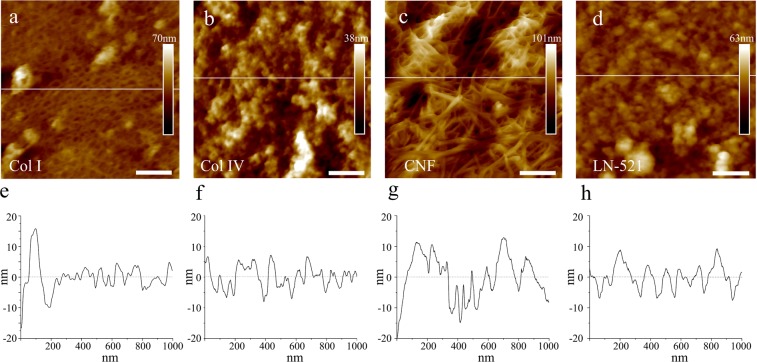
Representative AFM height images of biomaterial substrates formed by adsorption of the biomaterials on colloidal glass probes. The biomaterials used were (**a**) collagen I, (**b**) collagen IV, (**c**) CNF, (**d**) laminin-521 (scale bar is 200 nm). (**e**–**h**) Show the height topographic profiles corresponding to the lines marked in figures (**a**–**d**), respectively.

All the biomaterials were successfully adsorbed on the colloidal glass probes producing even and randomly spread coatings (Fig. 2). The uncoated glass spheres were probed as a control, and they exhibited a very different morphology with a surface roughness of 11.8 ± 0.5 nm (Fig. S1). A homogeneous film of Col I fibrils was formed on the colloidal probe surface, with a surface roughness of about 2.6 ± 0.5 nm **(**Fig. 2a). Col IV formed a coating with more variability in height and more globular-like morphology with the roughness of 5.5 ± 0.9 nm (Fig. 2b). The fibrils did not organize into large fibrillar structures as they do when deposited using Langmuir Schaefer deposition onto flat substrates^63,64^. The CNF coated probes showed clear fibrillar morphology with the highest roughness at 9.7 ± 2.9 nm (Fig. 2c). Lastly, laminin had a globular morphology with mean roughness around 4.9 ± 1.4 nm (Fig. 2d). All the coatings showed highly distinct morphology compared to the uncoated probe.

### Force interactions of cells and biomaterials

**Figure 3:**
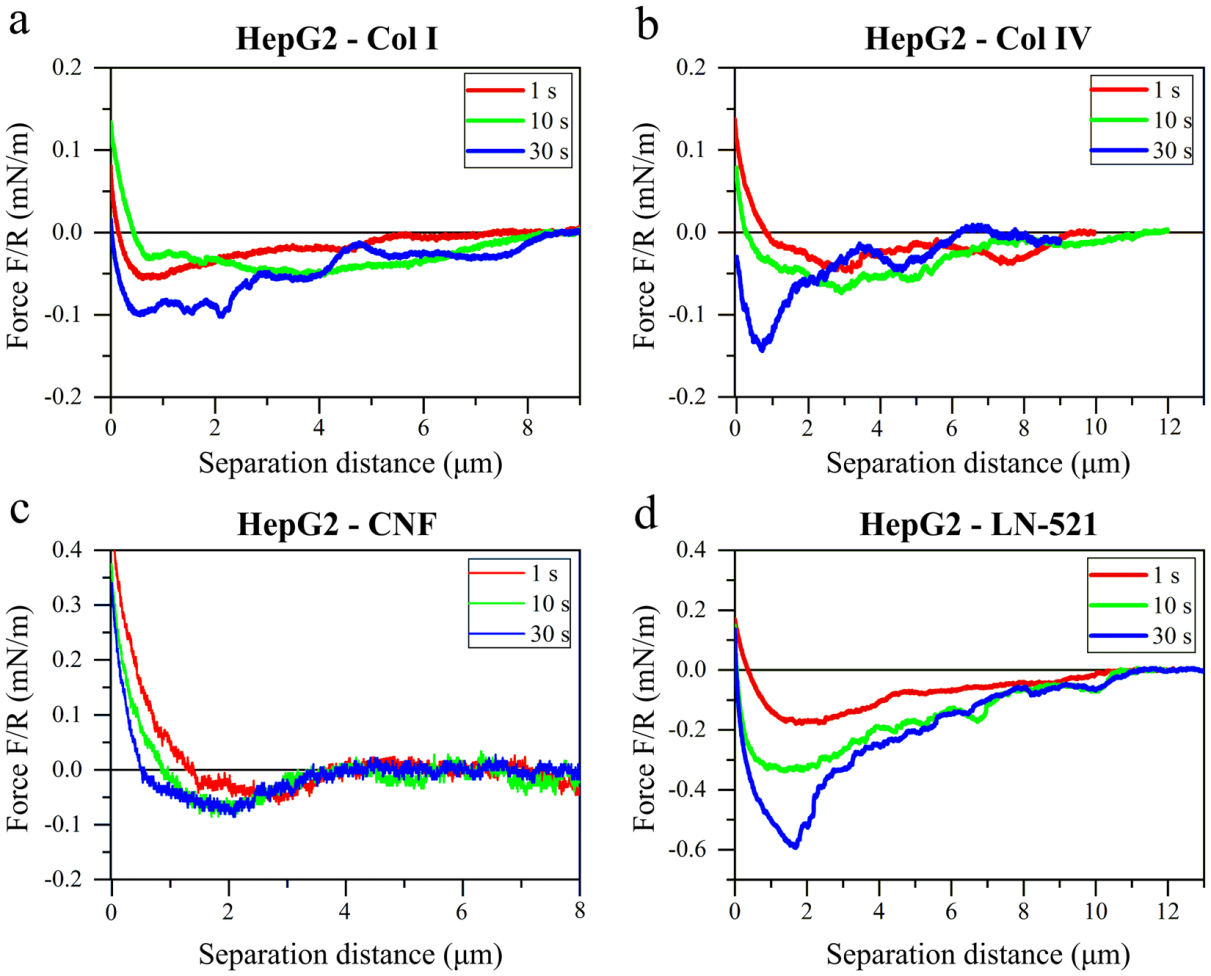
Retraction force curves between HepG2 cells and colloidal probes coated with: (**a**) collagen I, (**b**) collagen IV, (**c**) cellulose nanofibrils, and (**d**) laminin-521. Representative force curves are presented normalized by the probe radius R (Table S2) after different cells-probes contact times (1 s, 10 s, and 30 s).

**Figure 4:**
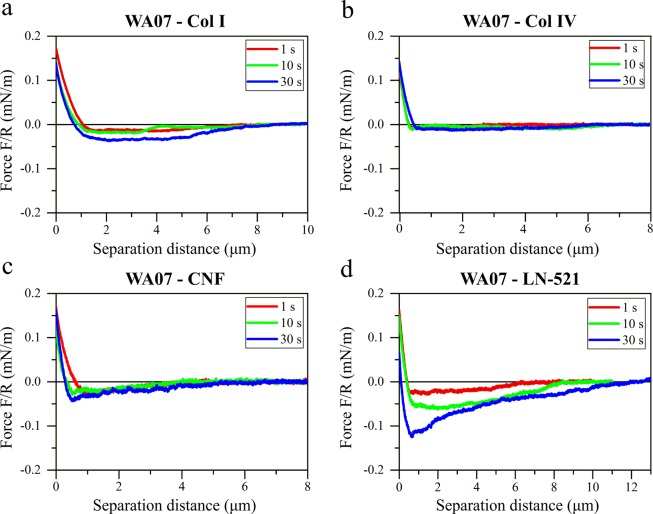
Retraction force curves between WA07 cells and colloidal probes coated with: (**a**) collagen I, (**b**) collagen IV, (**c**) cellulose nanofibrils, and (**d**) laminin-521. Representative force curves are presented normalized by the probe radius R (Table S2) after different cells-probes contact times (1 s, 10 s, and 30 s).

Figures 3 and 4 show representative retraction force curves between living cells and probes coated with different biomaterials. Control experiments with uncoated, PEI-coated, and APTES-coated probes are presented in the Supplementary Information (Fig. S3). The force interactions between studied biomaterials and two living cell types, HepG2 and WA07, exhibited a long-ranged adhesion, exceeding a separation distance of 6 µm for all materials except CNF (Figs 3 and 4). A clear contact-time dependent adhesion was observed for both cell lines with all the used biomaterials. The adhesion energy increased as the contact time increased.

The adhesion of collagens to HepG2 (Fig. 3a,b and Table S1) showed long range but moderate pull-off force. CNF showed significantly lower adhesion to this cell line than the other materials and the adhesion was also less dependent on the time in contact (Fig. 3c). On the contrary, the adhesion between LN-521 and HepG2 was very strong and contact-time dependent (Fig. 3d). The adhesion was significantly stronger compared to collagens, with a range that extended to separations over 10 µm. Interestingly, the force at zero distance was usually different for different contact times, a phenomenon that is related to the cell behavior. The cells and the colloidal probes were initially approached to contact until reaching a maximum applied load (the same during the experiment). Then cells and colloidal probes were kept in contact for different times before separating them. During the time in contact the cells could rearrange and flatten to some extent, provoking a change in the deflection of the colloidal probe-bearing cantilever (that is, a change in the force at zero distance) that was more evident as the contact time increased.

The selective nature of hPSCs was evident from the forces observed between the WA07 cells and the tested biomaterials. The cell affinity for Col I and Col IV was almost negligible, with only a notable adhesion after 30 s in contact (Fig. 4a,b). A low adhesion of CNF to hPSCs was observed after 30 s in contact, but that adhesion was still one of the weakest of all the tested systems, with a shorter range than for collagens (Fig. 4c). In contrast, the WA07 cells showed strong adhesive interactions to the LN-521-coated probes, and the highest pull-off force was observed for this system (Fig. 4d). Similarly as for HepG2 cells, the adhesion was dependent on the time in contact.

**Figure 5:**
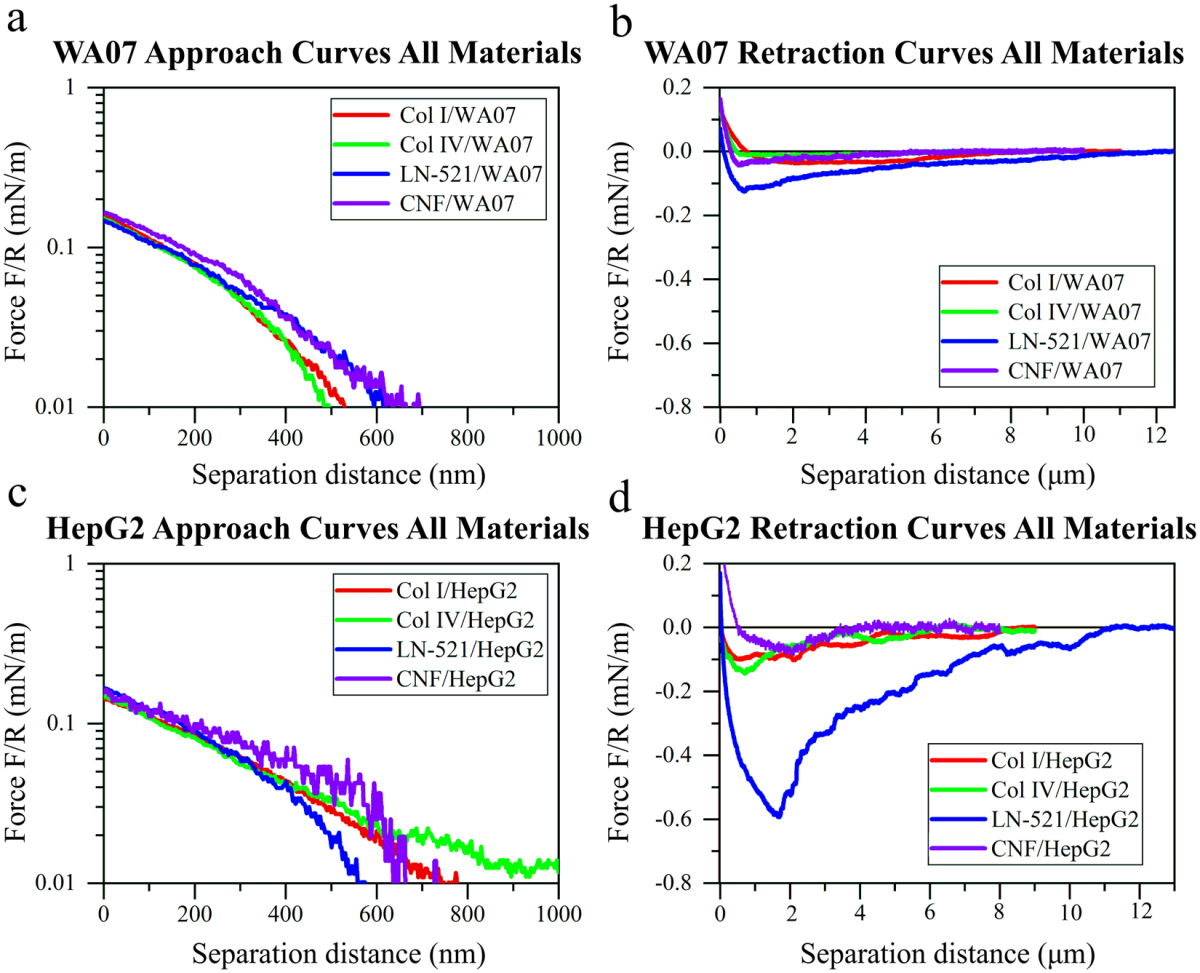
Representative normalized force curves between different biomaterial-coated probes and living cells: (**a**) approach curves in logarithmic scale and (**b**) retraction curves on WA07 cells; (**c**) approach curves in logarithmic scale and (**d**) retraction curves on HepG2 cells. The retraction curves were recorded after 30 s contact time between cells and probes. Force values were normalized by the probe radius *R* (Table S2).

**Figure 6:**
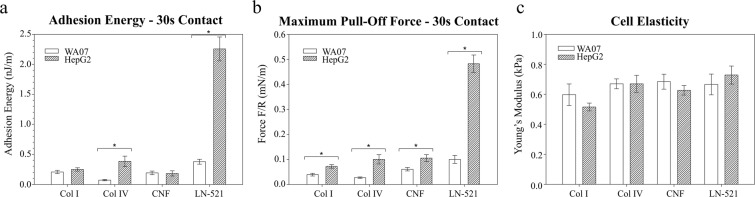
Comparison of adhesion energies (**a**), maximum pull-off forces (**b**), and cell elasticity (**c**) for HepG2 and WA07 cell interactions with collagen I (Col I), collagen IV (Col IV), cellulose nanofibrils (CNF), and laminin-521 (LN-521) at contact time of 30 s. Error bars are standard errors of mean and significant differences of p ≤ 0.05 are marked with *. Values were normalized by the probe radius *R*.

In Fig. 5 representative force curves upon approach (a, c) and retraction (b, d) are compared for the two cell lines. The forces upon approach were purely repulsive between biomaterials and both living cell types and were detected at a relative separation distances larger than 400 nm (Fig. 5a,c). At the high electrolyte concentration used, the electrostatic double layer repulsion would be very short-ranged and, therefore, the main source for the repulsion was the compression of the cell by the probe (consequently, the zero separation in the graphs actually corresponds to the point of maximum compression of the cells). The cell elasticity was estimated from the approach curves using the linearized Hertz model. Two different regimes with two values for the elastic modulus were observed when compressing the cells (Fig. S2). The first regime at lower applied force is usually ascribed to the compression of the membrane and molecular brushes (microvilli, microridges, glycocalyx), while the second regime at higher applied forces corresponds to the elasticity of the bulky cytosol of the cells^61^. In this work we report the elastic modulus of the cells corresponding to the second regime. In general, the obtained values indicated no statistical difference between the elasticity of HepG2 and WA07 cells (p > 0.05) (Fig. 6c). It must be considered that the biomaterials are also soft, and compression of biomaterial could partly contribute to the detected force. However, since the biomaterials form a thin layer on the glass probes, the compression of biomaterials would give rise to a short-range steric repulsion, suggesting that the long range of the force observed here is mainly dominated by the compression of the cells^55^. On the other hand, the comparison of the retraction force curves showed that the adhesion of biomaterials to HepG2 was in general stronger than to hPSCs after 30 s contact time (Fig. 5b,d).

Mean values of adhesion energies and pull-off forces for the cell-biomaterial systems studied in this work are presented in Fig. 6a,b and Table S1. The adhesion of Col I and Col IV to HepG2 cells was stronger than to WA07 cells. The adhesion was remarkably different for Col IV, with an adhesion energy about 5 times higher on HepG2 cells compared to WA07 cells (p ≤ 0.05) (Fig. 6a). For both cell lines, the strongest adhesion was observed with LN-521. Also in this case, the adhesion energy between HepG2 and LN-521 was remarkably higher (about six times higher) than between WA07 and LN-521 (p ≤ 0.05). In contrast, CNF showed similar low interactions with both cell types with no statistical difference. The maximum pull-off forces were generally in line with the adhesion energies (Fig. 6b), with the highest values obtained for cell-laminin interactions. No statistical differences between different measuring days or positions were noted with any system (p ≤ 0.05) (Fig. S4).

### Cell viability and cell-biomaterial interactions on cell cultures

**Figure 7:**
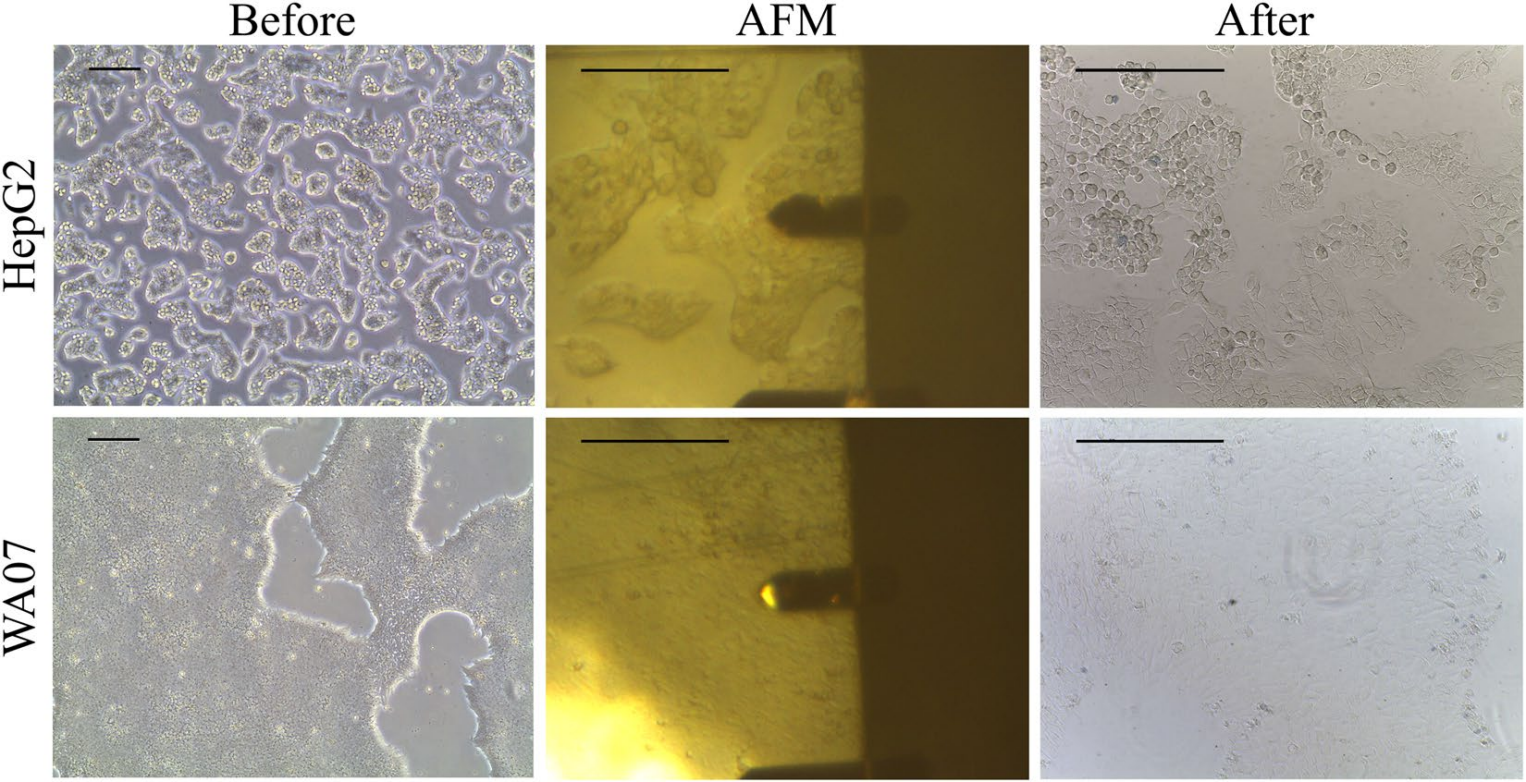
Representative images of HepG2 and WA07 cells before, during and after the force experiments. The cell viability after the experiments was controlled with Trypan Blue exclusion test (scale bars are 200 µm).

Care was taken to ensure that the cells were alive during the colloidal probe experiments. Hence, the measurements were conducted at +37 °C and the measurement time was kept short, under 1.5 h for WA07 and 2 h for HepG2 cells, due to the lack of CO_2_ control. The cells were constantly monitored with the AFM camera during the measurements (Fig. 7). After about 2 h in the AFM HepG2 and, especially, WA07 cells had a tendency at first to lose their cell-cell contacts, which affects the cell morphology, and finally to detach and die; hence it was easy to monitor cell viability *in situ* during experiments. This non-invasive cell viability test was used in order not to interrupt delicate cell-biomaterial interactions. For further confirmation of cell viability and to be able to test the cell condition right after the AFM measurements, Trypan Blue exclusion test with cell fixation was conducted. Trypan Blue exclusion test showed that the adherent cells had still excellent viability at the areas of measurements, which were the central area of the coverslips and cell colonies (Fig. 7).

**Figure 8:**
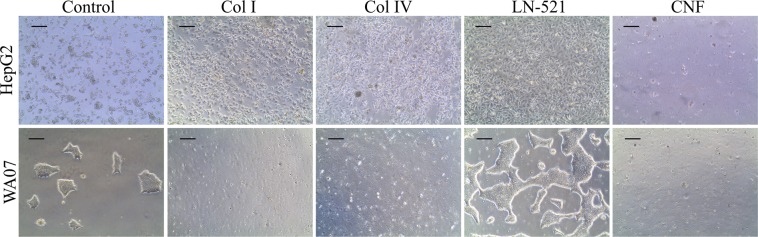
HepG2 and WA07 cells after 20 hours culture on a control matrix (plastic and Matrigel, respectively) and on test materials: collagen I, collagen IV, laminin-521 and cellulose nanofibrils (scale bars are 200 μm).

To validate the obtained cell interaction forces with the biomaterials, the studied cells were also cultured as 2D on the surfaces of the examined biomaterials. We performed three replicas for all systems. Cell culture on the biomaterials showed that HepG2 cells did not adhere to unmodified CNF and WA07 cells did not adhere to collagens or CNF after 20 hours incubation (Fig. 8). On the other hand, both cell lines showed great attachment to LN-521. The cell confluency was better with LN-521 than the control materials. Besides LN-521, collagens also showed higher HepG2 confluency compared to the controls. The cell morphology was affected by the culturing material; both cell lines showed ideal morphology, high viability and growth rate on LN-521 compared to other systems. Especially HepG2 showed typical hepatocyte-like cubic morphology on LN-521.

## Discussion

### Choice of biomaterials and force measurement set-up

The biomaterials used in this study have different properties concerning morphology, chemistry, and biological origin. Col I and unmodified natural CNF represent fibrillar materials (Fig. 2a,c, respectively), while Col IV and LN-521 have globular morphologies (Fig. 2b,d, respectively). The fibrils of Col I are thin and flexible and assemble as a smooth film on the probe, while the CNF forms a rougher fibril mat. Furthermore, CNF is sugar-based and hence differs from the other materials consisting of amino acids. CNF is also the only not ECM-derived material in this study. All these biomaterials have in common that they have been used for *in vitro* cell cultures and could be used in regenerative medicine. They were chosen for this work because they have different structural and chemical properties, and because they are xenobiotic-free, already in use in human cell culture applications, and commercially available. For comparison, we studied both widely used (Col I and Col IV) and novel (LN-521 and CNF) cell culture materials. All the biomaterials formed homogeneous, stable, and firmly attached films on the colloidal probes, with a similar morphology as reported for the materials in nature^10,63–66^.

The successful coating of the probes with different biomaterials and the stability of those coatings during force measurements were confirmed by AFM images (Fig. 2) and by comparison with control experiments using non-biomaterial coated probes (Fig. S3). PEI, a positively charged polymer, adheres strongly to cells^67,68^. For that reason, it has been used in single cell force spectroscopy to attach bacteria on cantilevers^69^. A strong adhesion between PEI and HepG2 cells was also observed in our control experiments. The adhesion was so strong that we had sometimes difficulties to reach the baseline, especially after 30 s contact time. PEI was used to coat the colloidal probes with CNF. The low adhesion observed between CNF-coated probes and cells clearly indicates that CNF fully covered the PEI-coated probe (as the AFM images also show, Fig. 2) and that the CNF coating was stable during the whole force experiments. We also observed that the adhesion of HepG2 cells to uncoated glass probes was significantly lower than to laminin-coated probes, confirming that laminin was firmly attached on the colloidal probe during the force measurements. The APTES layer under the biomaterial coating stabilizes the ECM protein coating and prevents well protein detachment in cell cultures^70^. APTES has shown stronger adhesive interactions with cells compared to glass but lower than fibronectin or poly-l-lysine^71^. APTES was used in our work to coat the colloidal probes with Col I and Col IV. Our control experiments reveal that the adhesion and/or force profiles were different between APTES-coated and collagen-coated probes against HepG2 cells.

Atomic force microscopy has proven its suitability for cell-biomaterial interaction studies. The most common set-up, SCFS with cells attached to the AFM probe, could not be used for our studies due to the choice of cell lines. hPSCs cannot survive as single cells on the AFM probes without the addition of Rock-inhibitor, which could disturb the measurements. The set-up of our choice, CPM, has been criticized because of the greater danger of bead contamination with cell remnants after contact^28,72^. We used relatively short contact times that reduce the probability of contamination, and most of the interactions were weak. In addition, we washed the colloidal probes with water or changed them if any suspicious of contamination was noticed during the measurements. Usually, no evident decay in adhesion or any other remarkable change in the recorded forces was observed when measured on the same position, which was considered as an indication of no contamination of the probe and stability of the biomaterial coating. On the other hand, CPM is faster and gentler than SCFS to the delicate cells, and thus a better option for the hPSCs studied here. With CPM, the cell status is easier to control during measurements compared to SCFS. This configuration also enables cell-cell contact thus providing a more natural environment for the cells and more correct cell polarization. It should also be noticed that, given the big size of our colloidal probes, the biomaterial-cell contact areas in our CPM experiments are in principle in the same range as in SCFS. Nevertheless, a direct comparison of the adhesion results obtained from both techniques is not straightforward since a precise determination of the contact areas (which affect the adhesion) is not easy.

Although cell-biomaterial interactions were quantified in this work using 2D cell cultures, the results can also be applied to 3D cultures. Cells in 3D structures like colonies, spheroids or tissue samples eventually interact with biomaterials through the surfaces of the cell assemblies; so similar forces are present regardless of the culturing method. The 3D configuration only increases the area in contact with the biomaterial. Nevertheless, different cell polarities in 2D and 3D cell cultures may result in some differences in cell-biomaterial interactions.

The presence of Ca^2+^ and Mg^2+^ ions in 1 × PBS+ play a critical role in controlling the activation of the cell adhesion molecules. In buffer without these cations, WA07 cells lose their cell-cell contacts within a few minutes followed by cell death and thus cannot be used for the studies. The activation of the most important cell-biomaterial adhesion molecules, integrins, are controlled by Ca^2+^ and Mg^2+^ ions, and thus they were included in the buffer^73^.

The adhesion of delicate cell lines such as hPSCs and biomaterials have not been studied with AFM before. Both hPSCs WA07 and HCC HepG2 cell lines were proven to be viable during and after the measurements, and thus the results can be considered reliable. The cells showed cell line specific behavior and morphology during the measurements and therefore can be viewed as representatives of their cell types.

### Effect of material properties on the cell interactions

The different nature of the biomaterials resulted in different force curve profiles within the same cell line (Fig. 5). Globular LN-521 had the highest pull-off force and largest magnitude of adhesion with both cell lines. There is a very fine saw-tooth pattern in the retraction curves, indicating step-wise breakage of bonds between cells and laminin. The strong affinity is explained by the presence of integrins in both cell types that interact with laminin as discussed in the following section. Both collagens, on the other hand, showed a long-ranged but low magnitude of adhesion, with considerably larger steps in the curve, that was most prominent in the case of interactions with HepG2 cells. This pattern could be due to collagen molecules being oriented into larger fibrillar structures, with both chemistry and morphology affecting the interaction pattern. Even though CNF has fibrillar morphology like collagens, the force profile was very different. The underlying reason is that the cells do not interact as strongly with polysaccharides as with proteins, so in this case, the interaction is dominated by the surface chemistry of the biomaterial.

Proteins from ECM interact with cells mainly through integrins found in the cell membranes. The interactions of sugars with cell membranes are weak and have scarcely been studied^74^. Our study shows a detailed quantification of the interactions of glucose-based CNF with the chosen cells. In accordance with previous findings measured with a different method and other cell types^74^, we observed that the un-normalized adhesion of a polysaccharide with cells was very weak, only about 2 nJ for WA07 and 2.7 nJ for HepG2 after 30 s in contact (Table S1e,f). A small pull-off force was detected at first, but the forces decayed to zero at much shorter distances compared to the protein-based substrates collagen and laminin. This is interesting to note since CNF has a similar fibrillary morphology as Collagen I. The corresponding un-normalized adhesion values for LN-521 interactions were 3.8 nJ for WA07 and 33 nJ for HepG2 (Table S1e,f). The two different collagens had very similar interactions with HepG2 cells, leading to the conclusion that the small differences in chemistry are not decisive for the total interactions with HCC cells. The forces between collagens and WA07 cell line showed slightly greater variability. On the other hand, the adhesive forces between WA07 cells and collagens were altogether very low (Fig. 6).

### Effect of cell lines on the interactions with biomaterials

The integrin-ECM protein complex is highly crucial for cell adhesion. Specific cell signaling can onset after integrin activation and complex formation. Integrins consist of several subtypes that are present in cell lines with specific combinations. Pluripotent hESCs predominantly express the laminin-binding integrin subunits α6 and β1, and the vitronectin binding αV and β5^75^. HepG2 cells express α2, α6, β1, and β4 integrin subunits^76^. From these, integrin subtype α2β1 binds collagens^76,77^. In other words, hPCSs do not have collagen specific integrins unlike HepG2 cells. But on the other hand, both these cell types have LN-521 binding integrins. In agreement with those studies, we observed significant adhesion only between those materials and cells that have the specific material binding integrin subtypes.

However, there are also non-integrin molecules that mediate cellular attachment. For instance, dystroglycans and syndecans are cell surface-associated molecules shown to interact directly with laminins. These interactions might be the reason for the strongest interactions of cells with laminin. Interactions of polysaccharides with cell membranes are weak and thus scarcely studied^74^. Here we present a detailed analysis of the interactions between HepG2 and WA07 cells and glucose-based CNF. The magnitude and profile of the force curves were similar, supporting the idea of similar, non-specific, interaction mechanisms of CNF, regardless of the cell type. The slightly higher pull-off forces between CNF and HepG2 cells could be explained by small differences in applied force.

The force curves measured on approach are also descriptive of the properties of cells and biomaterials. Those force curves showed a very long ranged repulsion due to the compression of both biomaterials and, especially, cells. The slightly longer range of the repulsion observed with HepG2 cells indicated that those cells were more compressible than WA07 cells. However, similar cell elasticities were obtained from the second regime of the approach force curves –corresponding to the compression of the bulky cytosol (Fig. S2)– for both cell lines. The differences observed in the onset of the repulsion on approach, ascribed to the compression of membrane features like microvilli, indicate that HepG2 and WA07 have different membrane structure as shown previously^78^. The obtained elasticity values were in line with previously reported values for cells^79,80^.

### Correlation between force measurements and *in vitro* cell culture tests

A clear correlation was observed when comparing the force experiment results with *in vitro* cell culture tests: weak adhesion between cells and biomaterials resulted in no cell attachment in *in vitro* cultures, whereas strong cell-biomaterial adhesion gave rise to good cell attachment in *in vitro* cell cultures (Fig. 8).

The cell morphology in *in vitro* cultures was also affected by the magnitude of the cell-biomaterial interaction. High adhesion to LN-521 resulted in higher confluency and more ideal cell morphology. Moderate adhesion of HepG2 to collagens provoked good attachment of cells compared to maintenance culture on plastic. Based on our results it can be seen that the magnitude of overall forces between cells and biomaterials results in higher cell attachment and better cell morphology. The critical point for cell attachment seemed to be a total adhesion energy of at least 0.23 nJ/m (Fig. 6a). Unlike adhesion energies, maximum pull-off forces do not follow a perfect correlation with cell attachment observations in *in vitro* cell cultures. The maximum pull-off force for the cells against unmodified CNF was of similar range as for collagens, but still, neither cell line grew on CNF coated substrates. However, the range of adhesion was considerably shorter for the CNF systems as compared to laminin or collagens, resulting in lower adhesion energy. These results suggest that for 2D cell culture, strong adhesion energy between cells and substrate is needed. We may conclude that CNF as such will not be a suitable 2D cell culture material. However, CNF has successfully been used for 3D cell culturing in CNF hydrogel.

Our findings lead to the conclusion that the negligible signaling of CNF with cells allows cell-cell contact to be dominant and thus allows the 3D spheroid formation inside the CNF hydrogel. It could be concluded, that low cell interactions with biomaterials may enhance spheroid formation and thus CNF hydrogel and suspension cultures enable excellent spheroid formation. In 3D cell cultures also scaffold stiffness and cell type affects the spheroid formation. CNF has shown to have concentration dependent and thus easily tunable stiffness. In addition, the negligible signaling between hPSCs and CNF could help these stem cells to remain undifferentiated for extended time periods as proven earlier by Lou *et al*.^13^. For some cases, it is beneficial to keep and carry the hPSC undifferentiated for further studies and culture expansion. In the case of stem cell differentiation, this might also be a disadvantage. Kanninen *et al*. showed that ECM components support the differentiation in the desired lineage^16^. From our force measurements, 3D scaffold could be eventually designed for suitable applications by surface modifying CNF hydrogel with more interacting molecules to improve cell-ECM interplay and controlled cell differentiation. However, to choose the appropriate modification strategy, the interactions between the different biomaterials need to be known. We recently showed that LN-521 has low affinity for CNF, but the addition of Col I besides LN-521 could be the suitable approach to functionalize CNF in different 2D and 3D cell culture applications^55^. We further note that although most previous literature state that cells do not adhere to CNF or have only negligible adhesion^15,81–84^, contradictive results have also been published. This may be both due to the difficulty to make conclusions about adhesion mechanisms based on indirect methods, and due to the variety of CNFs that have been used. Hence, direct force measurements with well-defined materials are more informative.

## Conclusions

We successfully probed the adhesive and repulsive interactions between different biomaterials (Col I, Col IV, LN-521, and CNF) and two cell lines never tested before, WA07 and HepG2, using CPM. The cells showed different binding affinities for the different biomaterials, with increasing adhesion energies as the contact time between cells and biomaterials increased. Both WA07 and, especially, HepG2 cells adhered the most strongly to LN-521, but they had low affinity for CNF. A notable adhesion was also observed between HepG2 cells and Col IV, although the corresponding adhesion energy was about five times smaller than in the case of LN-521. The respective force profiles showed that the detailed interactions were distinctively different. The results obtained from force experiments correlated with *in vitro* cell culture observations: cells successfully grew on those biomaterials on which they attached with adhesion energies above 0.23 nJ/m, indicating that cell-biomaterial interactions depend on biomaterial nature and cell characteristics. This work provides fundamental information to explain the mechanisms underlying cell behavior in *in vitro* cultures, the interaction of cancerous and normal cells with surrounding tissues, and the formation of cell spheroids in CNF hydrogels, among other cell phenomena.

## Supplementary information

Supplementary Information

## Data Availability

The datasets generated and/or analysed during the current study are not publicly available because they also form part of an ongoing study, but they are available from the corresponding authors on reasonable request.

## References

[1] Schlie-Wolter S, Ngezahayo A, Chichkov BN (2013). The selective role of ECM components on cell adhesion, morphology, proliferation and communication *in vitro*. Exp. Cell Res..

[2] Inman JL, Robertson C, Mott JD, Bissell MJ (2015). Mammary gland development: cell fate specification, stem cells and the microenvironment. Development.

[3] Kaukonen R (2016). Normal stroma suppresses cancer cell proliferation via mechanosensitive regulation of JMJD1a-mediated transcription. Nat. Commun..

[4] Lee EY, Parry G, Bissell MJ (1984). Modulation of secreted proteins of mouse mammary epithelial cells by the collagenous substrata. J. Cell Biol..

[5] Heydarkhan-Hagvall S (2008). Three-dimensional electrospun ECM-based hybrid scaffolds for cardiovascular tissue engineering. Biomaterials.

[6] Gieni RS, Hendzel MJ (2008). Mechanotransduction from the ECM to the genome: are the pieces now in place?. J. Cell. Biochem..

[7] Rao Pattabhi S, Martinez JS, Keller TC (2014). Decellularized ECM effects on human mesenchymal stem cell stemness and differentiation. Differentiation.

[8] Taubenberger AV, Hutmacher DW, Muller DJ (2014). Single-cell force spectroscopy, an emerging tool to quantify cell adhesion to biomaterials. Tissue Eng. Part B. Rev..

[9] Oryan A, Kamali A, Moshiri A, Baharvand H, Daemi H (2018). Chemical crosslinking of biopolymeric scaffolds: Current knowledge and future directions of crosslinked engineered bone scaffolds. Int. J. Biol. Macromol..

[10] Bhattacharya M (2012). Nanofibrillar cellulose hydrogel promotes three-dimensional liver cell culture. J. Control. Release.

[11] Malinen MM (2014). Differentiation of liver progenitor cell line to functional organotypic cultures in 3D nanofibrillar cellulose and hyaluronan-gelatin hydrogels. Biomaterials.

[12] Rinner B (2017). MUG-Mel2, a novel highly pigmented and well characterized NRAS mutated human melanoma cell line. Sci. Rep..

[13] Lou YR (2014). The use of nanofibrillar cellulose hydrogel as a flexible three-dimensional model to culture human pluripotent stem cells. Stem Cells Dev..

[14] Lauren P (2014). Technetium-99m-labeled nanofibrillar cellulose hydrogel for *in vivo* drug release. Eur. J. Pharm. Sci..

[15] Hakkarainen T (2016). Nanofibrillar cellulose wound dressing in skin graft donor site treatment. J. Control. Release.

[16] Kanninen LK (2016). Laminin-511 and laminin-521-based matrices for efficient hepatic specification of human pluripotent stem cells. Biomaterials.

[17] Humphries MJ (2001). Cell-substrate adhesion assays. Curr. Protoc. Cell. Biol..

[18] Kucik DF (2003). Measurement of adhesion under flow conditions. Curr. Protoc. Cell. Biol..

[19] Garcia AJ, Ducheyne P, Boettiger D (1997). Quantification of cell adhesion using a spinning disc device and application to surface-reactive materials. Biomaterials.

[20] Forrester JV, Lackie JM (1981). Effect of hyaluronic acid on neutrophil adhesion. J. Cell. Sci..

[21] Forrester JV, Lackie JM (1984). Adhesion of neutrophil leucocytes under conditions of flow. J. Cell. Sci..

[22] Sung KL, Sung LA, Crimmins M, Burakoff SJ, Chien S (1986). Determination of junction avidity of cytolytic T cell and target cell. Science.

[23] Kollmannsberger P, Fabry B (2007). High-force magnetic tweezers with force feedback for biological applications. Rev. Sci. Instrum..

[24] Walter N, Selhuber C, Kessler H, Spatz JP (2006). Cellular unbinding forces of initial adhesion processes on nanopatterned surfaces probed with magnetic tweezers. Nano Lett..

[25] Matthews BD (2004). Mechanical properties of individual focal adhesions probed with a magnetic microneedle. Biochem. Biophys. Res. Commun..

[26] Andersson M (2007). Using optical tweezers for measuring the interaction forces between human bone cells and implant surfaces: System design and force calibration. Rev. Sci. Instrum..

[27] Neuman KC, Nagy A (2008). Single-molecule force spectroscopy: optical tweezers, magnetic tweezers and atomic force microscopy. Nat. Methods.

[28] Friedrichs J (2013). A practical guide to quantify cell adhesion using single-cell force spectroscopy. Methods.

[29] Li F, Redick SD, Erickson HP, Moy VT (2003). Force measurements of the alpha5beta1 integrin-fibronectin interaction. Biophys. J..

[30] Taubenberger AV, Quent VM, Thibaudeau L, Clements JA, Hutmacher DW (2013). Delineating breast cancer cell interactions with engineered bone microenvironments. J. Bone Miner. Res..

[31] Yermolenko IS (2010). Origin of the nonadhesive properties of fibrinogen matrices probed by force spectroscopy. Langmuir.

[32] Ducker, W. A., Senden, T. J. & Pashley, R. M. Direct measurement of colloidal forces using an atomic force microscope. *Nature* **353**, 239–241 (1991).

[33] Muñoz Javier A (2006). Combined atomic force microscopy and optical microscopy measurements as a method to investigate particle uptake by cells. Small.

[34] Hagbard Louise, Cameron Katherine, August Paul, Penton Christopher, Parmar Malin, Hay David C., Kallur Therése (2018). Developing defined substrates for stem cell culture and differentiation. Philosophical Transactions of the Royal Society B: Biological Sciences.

[35] Pietilä M, Ivaska J, Mani SA (2016). Whom to blame for metastasis, the epithelial-mesenchymal transition or the tumor microenvironment?. Cancer Lett..

[36] Xu R, Boudreau A, Bissell MJ (2009). Tissue architecture and function: dynamic reciprocity via extra- and intra-cellular matrices. Cancer Metastasis Rev..

[37] Oikawa Y (2011). Melanoma cells produce multiple laminin isoforms and strongly migrate on α5 laminin(s) via several integrin receptors. Exp Cell Res..

[38] Lu P, Weaver VM, Werb Z (2012). The extracellular matrix: A dynamic niche in cancer progression. J Cell Biol..

[39] Pouliot N, Kusuma N (2013). Laminin-511: a multi-functional adhesion protein regulating cell migration, tumor invasion and metastasis. Cell Adh Migr..

[40] Gilkes DM, Semenza GL, Wirtz D (2014). Hypoxia and the extracellular matrix: drivers of tumour metastasis. Nat Rev Cancer..

[41] Pickup MW, Mouw JK, Weaver VM (2014). The extracellular matrix modulates the hallmarks of cancer. EMBO Rep..

[42] Barcus CE (2017). Elevated collagen-I augments tumor progressive signals, intravasation and metastasis of prolactin-induced estrogen receptor alpha positive mammary tumor cells. Breast Cancer Res..

[43] Qin Y, Rodin S, Simonson OE, Hollande F (2017). Laminins and cancer stem cells: Partners in crime?. Semin Cancer Biol..

[44] Ueda J (2018). Evaluation of the Impact of Preoperative Values of Hyaluronic Acid and Type IV Collagen on the Outcome of Patients with Hepatocellular Carcinoma After Hepatectomy. J Nippon Med Sch..

[45] Chen Dexin, Chen Gang, Jiang Wei, Fu Meiting, Liu Wenju, Sui Jian, Xu Shuoyu, Liu Zhangyuanzhu, Zheng Xiaoling, Chi Liangjie, Lin Dajia, Li Kai, Chen Weisheng, Zuo Ning, Lu Jianping, Chen Jianxin, Li Guoxin, Zhuo Shuangmu, Yan Jun (2019). Association of the Collagen Signature in the Tumor Microenvironment With Lymph Node Metastasis in Early Gastric Cancer. JAMA Surgery.

[46] Stolz DB, Michalopoulos GK (1997). Synergistic enhancement of EGF, but not HGF, stimulated hepatocyte motility by TGF-beta 1 *in vitro*. J. Cell. Physiol..

[47] Zheng X (2017). Collagen I promotes hepatocellular carcinoma cell proliferation by regulating integrin beta1/FAK signaling pathway in nonalcoholic fatty liver. Oncotarget.

[48] Rodin S (2014). Clonal culturing of human embryonic stem cells on laminin-521/E-cadherin matrix in defined and xeno-free environment. Nat. Commun..

[49] Cameron K (2015). Recombinant Laminins Drive the Differentiation and Self-Organization of hESC-Derived Hepatocytes. Stem Cell. Reports.

[50] Prockop DJ (1998). What holds us together? Why do some of us fall apart? What can we do about it?. Matrix Biol..

[51] Di Lullo GA, Sweeney SM, Korkko J, Ala-Kokko L, San Antonio JD (2002). Mapping the ligand-binding sites and disease-associated mutations on the most abundant protein in the human, type I collagen. J. Biol. Chem..

[52] Parkin JD (2011). Mapping structural landmarks, ligand binding sites, and missense mutations to the collagen IV heterotrimers predicts major functional domains, novel interactions, and variation in phenotypes in inherited diseases affecting basement membranes. Hum. Mutat..

[53] Beck K, Hunter I, Engel J (1990). Structure and function of laminin: anatomy of a multidomain glycoprotein. FASEB J..

[54] Aumailley M (2005). A simplified laminin nomenclature. Matrix Biol..

[55] Nugroho RWN (2019). Quantifying the interactions between biomimetic biomaterials –collagen I, collagen IV, laminin 521 and cellulose nanofibrils– by colloidal probe microscopy. Colloids Surf. B Biointerfaces.

[56] Goffin AJ, Rajadas J, Fuller GG (2010). Interfacial flow processing of collagen. Langmuir.

[57] Valle-Delgado JJ, Johansson LS, Osterberg M (2016). Bioinspired lubricating films of cellulose nanofibrils and hyaluronic acid. Colloids Surf. B Biointerfaces.

[58] Berger E, Vega N, Weiss-Gayet M, Geloen A (2015). Gene Network Analysis of Glucose Linked Signaling Pathways and Their Role in Human Hepatocellular Carcinoma Cell Growth and Survival in HuH7 and HepG2 Cell Lines. Biomed. Res. Int..

[59] Wadkin LE (2017). Dynamics of single human embryonic stem cells and their pairs: a quantitative analysis. Sci. Rep..

[60] Sader JE (1999). Calibration of rectangular atomic force microscope cantilevers. Rev. Sci. Instrum..

[61] Carl P, Schillers H (2008). Elasticity measurement of living cells with an atomic force microscope: data acquisition and processing. Pflugers Arch..

[62] Perry, S. W., Epstein, L. G. & Gelbard, H. A. In situ trypan blue staining of monolayer cell cultures for permanent fixation and mounting. *BioTechniques* **22**, 1020–1024 (1997). 10.2144/97226bm01 9187742

[63] Sorkio AE (2015). Biomimetic collagen I and IV double layer Langmuir-Schaefer films as microenvironment for human pluripotent stem cell derived retinal pigment epithelial cells. Biomaterials.

[64] Pastorino L (2014). Oriented collagen nanocoatings for tissue engineering. Colloids Surf. B Biointerfaces.

[65] Kim T, Kahng YH, Lee T, Lee K, Kim DH (2013). Graphene films show stable cell attachment and biocompatibility with electrogenic primary cardiac cells. Mol. Cells.

[66] Xia D (2016). The Ultrastructures and Mechanical Properties of the Descement’s Membrane in Fuchs Endothelial Corneal Dystrophy. Sci. Rep..

[67] Canale C, Petrelli A, Salerno M, Diaspro A, Dante S (2013). A new quantitative experimental approach to investigate single cell adhesion on multifunctional substrates. Biosens. Bioelectron..

[68] Vancha AR (2004). Use of polyethyleneimine polymer in cell culture as attachment factor and lipofection enhancer. BMC Biotechnol..

[69] Mulansky S, Saballus M, Friedrichs J, Bley T, Boschke E (2017). A novel protocol to prepare cell probes for the quantification of microbial adhesion and biofilm initiation on structured bioinspired surfaces using AFM for single-cell force spectroscopy. Eng. Life Sci..

[70] Masuda HT (2014). Coating extracellular matrix proteins on a (3-aminopropyl)triethoxysilane-treated glass substrate for improved cell culture. BioTechniques.

[71] Mao S (2018). Measurement of Cell-Matrix Adhesion at Single-Cell Resolution for Revealing the Functions of Biomaterials for Adherent Cell Culture. Anal. Chem..

[72] Friedrichs J, Helenius J, Muller DJ (2010). Quantifying cellular adhesion to extracellular matrix components by single-cell force spectroscopy. Nat. Protoc..

[73] Mould AP, Akiyama SK, Humphries MJ (1995). Regulation of integrin alpha 5 beta 1-fibronectin interactions by divalent cations. Evidence for distinct classes of binding sites for Mn^2+^, Mg^2+^, and Ca^2+^. J. Biol. Chem..

[74] Pincet F (2001). Ultraweak sugar-sugar interactions for transient cell adhesion. Biophys. J..

[75] Wang H, Luo X, Leighton J (2015). Extracellular Matrix and Integrins in Embryonic Stem Cell Differentiation. Biochem. Insights.

[76] Kawakami-Kimura N (1997). Involvement of hepatocyte growth factor in increased integrin expression on HepG2 cells triggered by adhesion to endothelial cells. Br. J. Cancer.

[77] Belkin AM, Stepp MA (2000). Integrins as receptors for laminins. Microsc. Res. Tech..

[78] Lou YR (2015). Silica bioreplication preserves three-dimensional spheroid structures of human pluripotent stem cells and HepG2 cells. Sci Rep..

[79] Kiss R (2011). Elasticity of human embryonic stem cells as determined by atomic force microscopy. J. Biomech. Eng..

[80] Ofek G (2009). Mechanical characterization of differentiated human embryonic stem cells. J. Biomech. Eng..

[81] Courtenay, J. C., Sharma, R. I. & Scott, J. L. Recent Advances in Modified Cellulose for Tissue Culture Applications. *Molecules* **23**, 654 (2018). 10.3390/molecules23030654 PMC6017284 29538287

[82] Courtenay James C., Deneke Christoph, Lanzoni Evandro M., Costa Carlos A., Bae Yongho, Scott Janet L., Sharma Ram I. (2017). Modulating cell response on cellulose surfaces; tunable attachment and scaffold mechanics. Cellulose.

[83] Taokaew S, Phisalaphong M, Newby BZ (2015). Modification of bacterial cellulose with organosilanes to improve attachment and spreading of human fibroblasts. Cellulose.

[84] Fu L, Zhang J, Yang G (2013). Present status and applications of bacterial cellulose-based materials for skin tissue repair. Carbohydr. Polym..

