# Author Correction: Quantified forces between HepG2 hepatocarcinoma and WA07 pluripotent stem cells with natural biomaterials correlate with *in vitro* cell behavior

**DOI:** 10.1038/s41598-020-65140-8

**Published:** 2020-05-26

**Authors:** Riina Harjumäki, Robertus Wahyu N. Nugroho, Xue Zhang, Yan-Ru Lou, Marjo Yliperttula, Juan José Valle-Delgado, Monika Österberg

**Affiliations:** 1 Department of Bioproducts and Biosystems, School of Chemical Engineering, Aalto University, FI-00076 Aalto, Finland; 2 Division of Pharmaceutical Biosciences, Faculty of Pharmacy, University of Helsinki, FI-00014 Helsinki, Finland; 3 Department of Pharmaceutical and Pharmacological Sciences, University of Padova, I-35131 Padova, Italy

Correction to: *Scientific Reports* 10.1038/s41598-019-43669-7, published online 14 May 2019

This Article contains two errors in the Discussion section under the subheading ‘Effect of material properties on the cell interactions’.

“In accordance with previous findings measured with a different method and other cell types^74^, we observed that the un-normalized adhesion of a polysaccharide with cells was very weak, only about 2 nJ for WA07 and 2.7 nJ for HepG2 after 30 s in contact (Table S1e,f)”

should read:

“In accordance with previous findings measured with a different method and other cell types^74^, we observed that the un-normalized adhesion of a polysaccharide with cells was very weak, only about 1.6 fJ for WA07 and 2.7 fJ for HepG2 after 30 s in contact (Table S1e,f)”.

Secondly,

“The corresponding un-normalized adhesion values for LN-521 interactions were 3.8 nJ for WA07 and 33 nJ for HepG2 (Table S1e,f)”

should read:

“The corresponding un-normalized adhesion values for LN-521 interactions were 3.8 fJ for WA07 and 33 fJ for HepG2 (Table S1e,f)”.

Additionally, the Supplementary Information that accompanies this Article contains errors in Table S1, S2 and S3.

In Table S1, the units of adhesion energy are incorrectly given as nJ instead of fJ. Additionally, three values of adhesion energy for CNF in panels (b), (d) and (f) are incorrect.

In Table S2, two values of radius are incorrect for CNF in panel (a) and Col I in panel (b).

In Table S3, the units of adhesion energy are incorrectly given as nJ instead of fJ.

The correct Tables S1, S2 and S3 appear below as Tables 1–3, respectively.

**Table 1:** Adhesion energies and maximum pull-off forces for HepG2 (a,c,e) and WA07 (b,d,f) cell interactions with collagen I (Col I), collagen IV (Col IV), cellulose nanofibrils (CNF), and laminin-521 (LN-521) at contact times of 1, 10 and 30 seconds. Mean values and standard errors of the mean (SEM) are shown.

a	HepG2 – 1 s contact						b	WA07 – 1 s contact					
	Number of force curves analyzed	Normalized adhesion energy (nJ/m)	SEM (nJ/m)	Adhesion energy (fJ)	Maximum pull-off force (mN/m)	SEM (mN/m)		Number of force curves analyzed	Normalized adhesion energy (nJ/m)	SEM (nJ/m)	Adhesion energy (fJ)	Maximum pull-off force (mN/m)	SEM (mN/m)
Col I	37	0.168	0.027	2.54	0.051	0.008	Col I	10	0.060	0.008	0.88	0.016	0.002
Col IV	21	0.319	0.042	4.32	0.074	0.008	Col IV	20	0.026	0.004	0.32	0.013	0.002
CNF	24	0.122	0.026	1.79	0.085	0.012	CNF	48	0.098	0.012	1.00	0.032	0.002
LN-521	23	0.94	0.14	13.7	0.199	0.025	LN-521	48	0.231	0.038	2.19	0.053	0.007
**c**	**HepG2 – 10 s contact**						**d**	**WA07 – 10 s contact**					
	**Number of force curves analyzed**	**Normalized adhesion energy (nJ/m)**	**SEM (nJ/m)**	**Adhesion energy (fJ)**	**Maximum pull-off force (mN/m)**	**SEM (mN/m)**		**Number of force curves analyzed**	**Normalized adhesion energy (nJ/m)**	**SEM (nJ/m)**	**Adhesion energy (fJ)**	**Maximum pull-off force (mN/m)**	**SEM (mN/m)**
Col I	29	0.213	0.019	3.24	0.069	0.007	Col I	11	0.051	0.012	0.75	0.015	0.003
Col IV	13	0.374	0.056	5.08	0.090	0.010	Col IV	21	0.075	0.013	0.91	0.030	0.004
CNF	24	0.153	0.030	2.25	0.097	0.014	CNF	44	0.130	0.016	1.33	0.044	0.004
LN-521	20	1.15	0.14	16.8	0.240	0.027	LN-521	29	0.317	0.042	3.14	0.066	0.006
**e**	**HepG2 – 30 s contact**						**f**	**WA07 – 30 s contact**					
	**Number of force curves analyzed**	**Normalized adhesion energy (nJ/m)**	**SEM (nJ/m)**	**Adhesion energy (fJ)**	**Maximum pull-off force (mN/m)**	**SEM (mN/m)**		**Number of force curves analyzed**	**Normalized adhesion energy (nJ/m)**	**SEM (nJ/m)**	**Adhesion energy (fJ)**	**Maximum pull-off force (mN/m)**	**SEM (mN/m)**
Col I	22	0.249	0.028	3.90	0.074	0.006	Col I	12	0.207	0.026	3.04	0.039	0.005
Col IV	11	0.384	0.087	5.27	0.090	0.014	Col IV	22	0.072	0.011	0.87	0.027	0.003
CNF	26	0.182	0.043	2.68	0.045	0.014	CNF	46	0.160	0.026	1.64	0.055	0.006
LN-521	18	2.26	0.20	33.0	0.482	0.035	LN-521	27	0.379	0.041	3.76	0.100	0.016

**Table 2:** Radii (µm) of the used colloidal probes for force measurements between HepG2 (a) and WA07 (b) cells and collagen I (Col I), collagen IV (Col IV), cellulose nanofibrils (CNF), and laminin-521 (LN-521).

a			b		
HepG2	Col I	13.0 – 19.2	WA07	Col I	14.7 – 17.6
	Col IV	13.5 – 14.4		Col IV	12.2
	CNF	14.7		CNF	10.3
	LN-521	14.6		LN-521	8.2 – 10.2

**Table 3:** Adhesion energies and maximum pull-off forces for HepG2 cell interactions with uncoated, APTES-coated, and PEI-coated glass probes after 1 s (a), 10 s (b), and 30 s (c) contact times. The radii of used colloidal probes (µm) are presented in (d). Mean values and standard errors of the mean (SEM) are shown.

**a**	**HepG2 – controls, 1 s contact**						**b**	**HepG2 – controls, 10 s contact**					
	**Number of force curves analyzed**	**Normalized adhesion energy (nJ/m)**	**SEM (nJ/m)**	**Adhesion energy (fJ)**	**Maximum pull-off force (mN/m)**	**SEM (mN/m)**		**Number of force curves analyzed**	**Normalized adhesion energy (nJ/m)**	**SEM (nJ/m)**	**Adhesion energy (fJ)**	**Maximum pull-off force (mN/m)**	**SEM (mN/m)**
Glass	18	0.023	0.007	0.36	0.011	0.002	Glass	17	0.030	0.007	0.46	0.015	0.002
APTES	12	0.043	0.008	0.56	0.018	0.002	APTES	6	0.090	0.042	1.18	0.042	0.013
PEI	7	0.66	0.19	7.22	0.150	0.048	PEI	5	0.45	0.19	4.88	0.086	0.027
**c**	**HepG2 – controls, 30 s contact**						d	Radii (µm) of control probes					
	Number of force curves analyzed	Normalized adhesion energy (nJ/m)	SEM (nJ/m)	Adhesion energy (fJ)	Maximum pull-off force (mN/m)	SEM (mN/m)							
Glass	15	0.083	0.020	1.30	0.032	0.005		HepG2	Glass	15.6			
APTES	7	0.172	0.026	2.24	0.124	0.026			APTES	13.1-13.5			
PEI	7	1.14	0.32	12.5	0.31	0.11			PEI	11.0-13.4			

