# Supplementary material for: Quantified forces between HepG2 hepatocarcinoma and WA07 pluripotent stem cells with natural biomaterials correlate with in vitro cell behavior
Source: Sci Rep. 2019 May 14;9:7354. doi: 10.1038/s41598-019-43669-7 (PMC6517585; doi:10.1038/s41598-019-43669-7)
Supplement: Supplementary file 1 — Supplementary Information [file 41598_2019_43669_MOESM1_ESM.pdf]

Supplementary Information

Quantified forces between HepG2 hepatocarcinoma and WA07 pluripotent stem cells with natural biomaterials correlate with *in vitro* cell behavior

Riina Harjumäki[1,2], Robertus Wahyu N. Nugroho[1], Xue Zhang[1], Yan-Ru Lou[2], Marjo Yliperttula[2,3], Juan José Valle-Delgado[1], Monika Österberg[1*]*

[1]*Department of Bioproducts and Biosystems, School of Chemical Engineering, Aalto University, FI-00076 Aalto, Finland*

[2]*Division of Pharmaceutical Biosciences, Faculty of Pharmacy, University of Helsinki, FI-00014 Helsinki, Finland*

[3]*Department of Pharmaceutical and Pharmacological Sciences, University of Padova, I-35131 Padova, Italy*

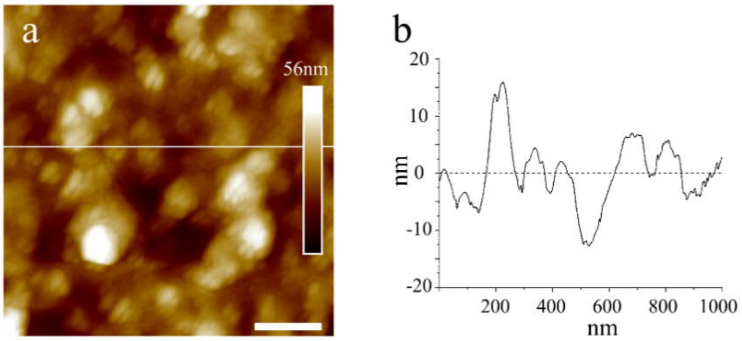

Figure S1. High-resolution image of an uncoated glass colloidal probe: (a) AFM height image (scale bar 200 nm); (b) topographic profile corresponding to the line marked in (a).

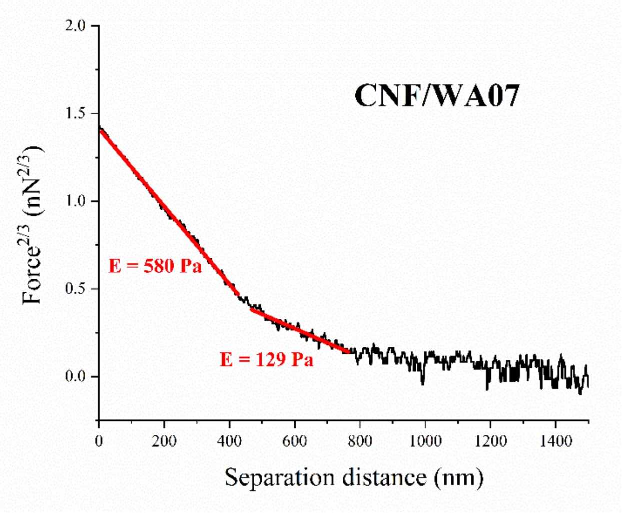

Figure S2. Analysis of WA07 cell elasticity from a representative approach force curve obtained when measuring the interaction between WA07 cells and cellulose nanofibrils (CNF).

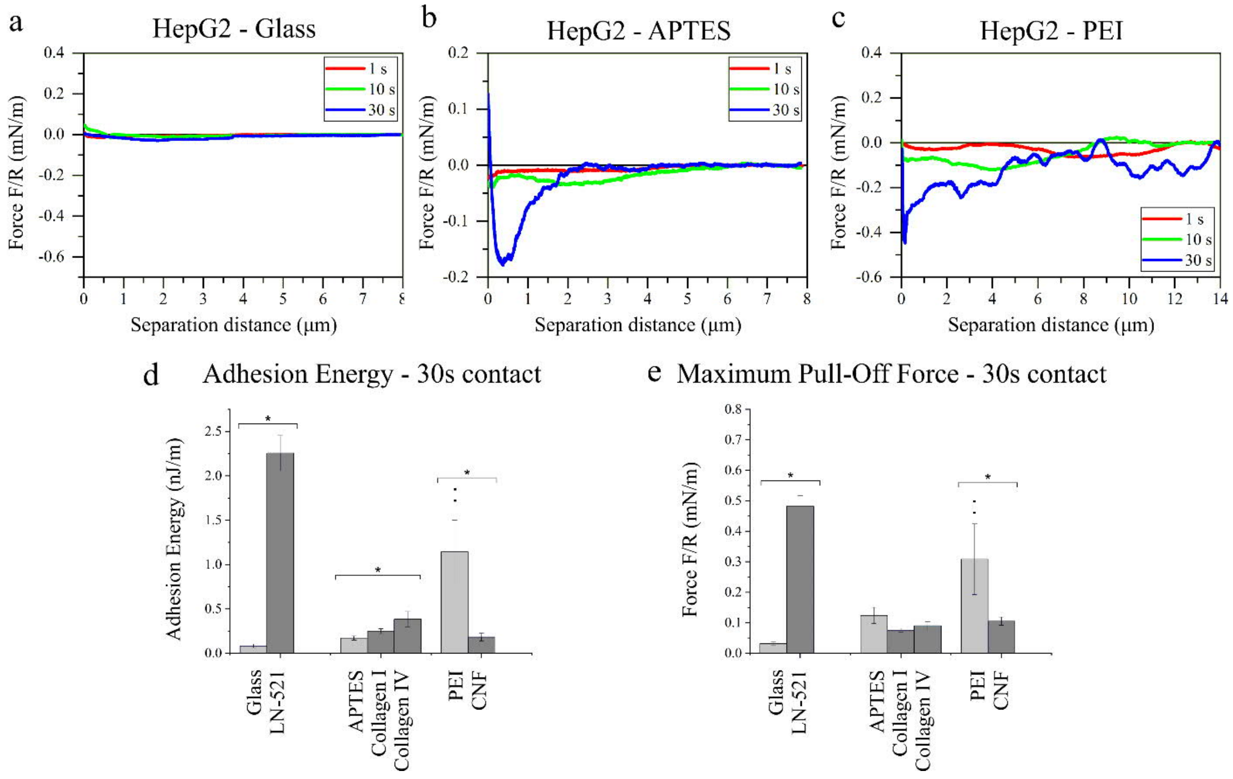

Figure S3. Retraction force curves, adhesion energy and maximum pull-off forces between HepG2 cells and control probes. Representative force curves for (a) uncoated glass probe and probes coated with (b) (3-aminopropyl)triethoxysilane (APTES) and (c) polyethyleneimine (PEI) are presented normalized by the probe radius *R* (Table S3) after different cells-probes contact times (1 s, 10 s, and 30 s). Comparison of adhesion energies (d) and maximum pull-off forces (e) at contact time of 30 s. Error bars are standard errors of mean and significant differences of $p \leq 0.05$ are marked with *. Values were normalized by the probe radius *R*.

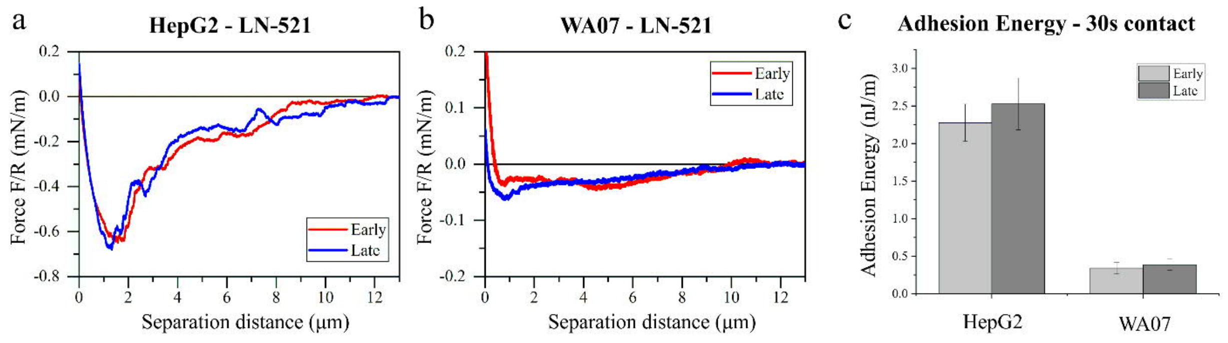

Figure S4. Examples of measurement reproducibility for the interactions between HepG2 and WA07 cells with LN-521 after 30 s contact time. Representative force curves at different locations of the same cell plate are presented for (a) HepG2 and (b) WA07, normalized by the probe radius *R*. Force curves at the first location (early) were obtained within the first 45 min or 30 min of the experiment for HepG2 and WA07, respectively. Force curves at the second location (late) were obtained within the last hour of the experiment (experiment duration up to 2 h or 1.5 h for HepG2 and WA07, respectively). Mean values of the corresponding adhesion energy for all the force curves obtained at the first (early) and second (late) locations are shown in (c), normalized by the probe radius *R*. Error bars are standard errors of mean. No significant differences ($p > 0.05$) were observed between early and late results.

Table S1. Adhesion energies and maximum pull-off forces for HepG2 (a,c,e) and WA07 (b,d,f) cell interactions with collagen I (Col I), collagen IV (Col IV), cellulose nanofibrils (CNF), and laminin-521 (LN-521) at contact times of 1, 10 and 30 seconds. Mean values and standard errors of the mean (SEM) are shown.

a

HepG2 - 1s Contact

	Number of force curves analyzed	Normalised adhesion energy (nJ/m)	SEM (nJ/m)	Adhesion energy (nJ)	Maximum pull-off force (mN/m)	SEM (mN/m)
Col I	37	0.168	0.027	2.54	0.051	0.008
Col IV	21	0.319	0.042	4.32	0.074	0.008
CNF	24	0.122	0.026	1.79	0.085	0.012
LN-521	23	0.938	0.138	13.7	0.199	0.025

b

WA07 - 1s Contact

	Number of force curves analyzed	Normalised adhesion energy (nJ/m)	SEM (nJ/m)	Adhesion energy (nJ)	Maximum pull-off force (mN/m)	SEM (mN/m)
Col I	10	0.06	0.008	0.88	0.016	0.002
Col IV	20	0.026	0.004	0.319	0.013	0.002
CNF	48	0.098	0.012	1.2	0.032	0.002
LN-521	48	0.231	0.038	2.19	0.053	0.007

c

HepG2 - 10s Contact

	Number of force curves analyzed	Normalised adhesion energy (nJ/m)	SEM (nJ/m)	Adhesion energy (nJ)	Maximum pull-off force (mN/m)	SEM (mN/m)
Col I	29	0.213	0.019	3.24	0.069	0.007
Col IV	13	0.374	0.056	5.08	0.09	0.01
CNF	24	0.153	0.03	2.25	0.097	0.014
LN-521	20	1.151	0.14	16.8	0.24	0.027

d

WA07 - 10s Contact

	Number of force curves analyzed	Normalised adhesion energy (nJ/m)	SEM (nJ/m)	Adhesion energy (nJ)	Maximum pull-off force (mN/m)	SEM (mN/m)
Col I	11	0.051	0.012	0.747	0.015	0.003
Col IV	21	0.075	0.013	0.91	0.03	0.004
CNF	44	0.13	0.016	1.43	0.044	0.004
LN-521	29	0.317	0.042	3.14	0.066	0.006

e

HepG2 - 30s Contact

	Number of force curves analyzed	Normalised adhesion energy (nJ/m)	SEM (nJ/m)	Adhesion energy (nJ)	Maximum pull-off force (mN/m)	SEM (mN/m)
Col I	22	0.249	0.028	3.9	0.074	0.006
Col IV	11	0.384	0.087	5.27	0.09	0.014
CNF	26	0.182	0.043	2.68	0.045	0.014
LN-521	18	2.256	0.199	33.0	0.482	0.035

f

WA07 - 30s Contact

	Number of force curves analyzed	Normalised adhesion energy (nJ/m)	SEM (nJ/m)	Adhesion energy (nJ)	Maximum pull-off force (mN/m)	SEM (mN/m)
Col I	12	0.207	0.026	3.039	0.039	0.005
Col IV	22	0.072	0.011	0.874	0.027	0.003
CNF	46	0.16	0.026	1.96	0.055	0.006
LN-521	27	0.379	0.041	3.76	0.1	0.016

Table S2. Radii (µm) of the used colloidal probes for force measurements between HepG2 (a) and WA07 (b) cells and collagen I (Col I), collagen IV (Col IV), cellulose nanofibrils (CNF), and laminin-521 (LN-521).

a

HepG2	Col I	13.0 - 19.2
	Col IV	13.5 - 14.4
	CNF	10.3
	LN-521	14.6

b

WA07	Col I	14.7
	Col IV	12.2
	CNF	10.3
	LN-521	8.2 - 10.2

Table S3. Adhesion energies and maximum pull-off forces for HepG2 cell interactions with uncoated, APTES-coated, and PEI-coated glass probes after 1 s (a), 10 s (b), and 30 s (c) contact times. The radii of used colloidal probes (µm) are presented in (d). Mean values and standard errors of the mean (SEM) are shown.

a

HepG2 - Controls 1s Contact

	Number of force curves analyzed	Normalised adhesion energy (nJ/m)	SEM (nJ/m)	Adhesion energy (nJ)	Maximum pull-off force (mN/m)	SEM (mN/m)
Glass	18	0.023	0.007	0.363	0.011	0.002
APTES	12	0.043	0.008	0.561	0.018	0.002
PEI	7	0.659	0.192	7.22	0.150	0.048

b

HepG2 - Controls 10s Contact

	Number of force curves analyzed	Normalised adhesion energy (nJ/m)	SEM (nJ/m)	Adhesion energy (nJ)	Maximum pull-off force (mN/m)	SEM (mN/m)
Glass	17	0.030	0.007	0.460	0.015	0.002
APTES	6	0.090	0.042	1.175	0.042	0.013
PEI	5	0.446	0.193	4.88	0.086	0.027

c

HepG2 - Controls 30s Contact

	Number of force curves analyzed	Normalised adhesion energy (nJ/m)	SEM (nJ/m)	Adhesion energy (nJ)	Maximum pull-off force (mN/m)	SEM (mN/m)
Glass	15	0.083	0.020	1.30	0.032	0.005
APTES	7	0.172	0.026	2.24	0.124	0.026
PEI	7	1.143	0.320	12.52	0.309	0.105

d

Radii of Control Probes

HepG2	Glass probe	15.6
	APTES	13.1-13.5
	PEI	11.0-13.4
